# Epidemic of Vitamin D Deficiency and Its Management: Awareness among Indian Medical Undergraduates

**DOI:** 10.1155/2017/2517207

**Published:** 2017-04-03

**Authors:** Yangshen Lhamo, Preeta Kaur Chugh, Sandhya R. Gautam, C. D. Tripathi

**Affiliations:** ^1^Department of Pharmacology, North DMC Medical College & Hindu Rao Hospital, New Delhi, India; ^2^Department of Pharmacology, Vardhman Mahavir Medical College & Safdarjung Hospital, New Delhi, India

## Abstract

Vitamin D deficiency is prevalent across all age groups in epidemic proportions. The purpose of this study was to acquire a baseline assessment and create awareness among medical students regarding vitamin D. A cross-sectional, voluntary survey was conducted among undergraduate medical students. Data were collected using a questionnaire which assessed the level of knowledge students had with regard to where vitamin D comes from, what it does for health, how much is recommended, factors that affect its levels, and deficiency management. Majority of students were unaware that vitamin D deficiency has attained epidemic proportions. Though bone and skeletal disorders as a complication of vitamin D deficiency were known, a large number were unaware of systemic consequences (diabetes mellitus, cardiovascular diseases, and cancers). Only one-third of respondents were aware of duration and timing of sun exposure required for adequate serum vitamin D levels. However, we observed lack of awareness among students regarding the various biochemical forms, dose, and duration of vitamin D supplementation for treatment of nutritional deficiency. Our study highlighted a lack of knowledge about the importance of vitamin D, worldwide prevalence of vitamin D deficiency, and its management among medical students. Promoting vitamin D health awareness, if replicated across populations, could lead to positive health outcomes globally.

## 1. Introduction

Vitamin D deficiency is a worldwide epidemic and yet, it is a problem that is largely unknown by majority of population [1]. Widespread prevalence in all age groups including toddlers, school children, men, women, elderly, pregnant women, and their neonates in both rural and urban areas has been documented [2]. Young adults are also potentially at high-risk for vitamin D deficiency. During childhood, this deficiency can cause growth retardation and skeletal deformities, while in adults, muscle weakness and fractures may ensue [3, 4]. In addition to its importance for bone health, recent evidence suggests that vitamin D is also useful in promoting cardiovascular health and preventing chronic diseases (diabetes mellitus, autoimmune disorders, and various cancers) [5, 6].

One of the major reasons for the worldwide spread of this nutritional disorder has been lack of awareness about the importance of vitamin D, its health benefits, and prevention of deficient states across populations [7–9]. It has been suggested that awareness and educational campaigns about vitamin D at the community level targeting both general and high-risk populations could help prevent long-term health consequences [10]. Primary education targeting younger populations is known to increase the likelihood of positive health behaviour that persists throughout and protect from disease development and progression later in life [11, 12]. Apart from targeting young adults at the community level, young medical students also need to be a focus for group interventions, as staggeringly low rates of awareness have been reported among them [13]. Selection of young medical students in this study provides a twofold opportunity of targeting a section of the general population that would also be future health providers of the community. Medical students are an integral part of future health-related progress of the community at large and should therefore be the target for inducing such long-term changes [12]. This next generation of professionals could influence the progression of future health education programs, policy development, and formation of social norms and beliefs about health and health promoting behaviours [14]. It is crucial to have a thorough understanding of student's motivation, knowledge, and attitudes towards a health behaviour before being able to create effective and targeted health promotion programs [15, 16]. Gaining a baseline understanding of medical students' current vitamin D related knowledge may be first step in programme development and is the purpose of this study. The findings from this assessment will provide important information to determine what next steps will be necessary to promote sufficient levels of vitamin D among medical students and health professionals. Thus, this study is an attempt for sensitization of medical undergraduates early in their training regarding vitamin D and prevention and treatment of its deficiency.

## 2. Materials and Methods

### 2.1. Study Design and Settings

Our study was conducted among the MBBS undergraduate students about one and half year after enrolment in medical school, at the Department of Pharmacology, Vardhman Mahavir Medical College (VMMC) and associated Safdarjung Hospital, New Delhi, India. This research was conducted as a cross-sectional, voluntary survey over a period of one and a half year. The study was approved by the Institutional Ethics Committee of Vardhman Mahavir Medical College and Safdarjung Hospital, New Delhi, India.

### 2.2. Data Collection and Questionnaire

The questionnaire was given to 300 undergraduate medical students. Before filling the questionnaire, necessary information was given by the researchers. The participants were allowed to get their doubts cleared. Data were collected using a questionnaire composed of two parts. First part included questions on age and gender and second half focused on vitamin D. The main objective of the study was to assess vitamin D knowledge among medical students. The 16-question, predominantly multiple-choice survey took approximately 15 minutes or less to complete. The survey questions assessed the level of knowledge students had with regard to where vitamin D comes from, what it does for health, how much is recommended, factors that affect vitamin D levels, and the prevalence of vitamin D deficiency. In our study, apart from questions on vitamin D deficiency and its management, two questions focused on sun exposure (duration and timing) in New Delhi, India, wherein the study was conducted (Table 1).

Validation of questionnaire to assess reproducibility and suitability was assessed using a small pretest in 10 subjects. The pretest participants were then contacted for their feedback and understanding with respect to the questionnaire. The Cronbach alpha was estimated to be 0.7, suggesting good internal consistency and an overall reliability. All student data were collected anonymously and deidentified by a member of staff not involved in the assessment of this cohort. After completion of the questionnaire, the students were taught about vitamin D, its importance, vitamin D deficiency, and its management in a lecture to create knowledge base.

### 2.3. Data Analysis

Statistical analysis was carried out using SPSS. Descriptive statistics were used to characterize the population and to identify the frequencies of participant's knowledge of vitamin D deficiency. A *p* < 0.05 was considered significant.

## 3. Results

### 3.1. Knowledge of Importance of Vitamin D and Its Deficiency

Only 17.8% of participants correctly identified that vitamin D deficiency has achieved epidemic proportions, while less than half of the students believed that it is prevalent only in urban population (47.2%) or limited to high-risk groups (23.8%). Majority of the participants (76.1%) correctly identified infants, pregnant, and lactating women as high-risk groups. However, they were not aware (92%) that elderly and diabetics were also at a greater risk to develop vitamin D deficiency than the general population. Though bone and skeletal disorders as a complication of deficiency were known (94.4%), majority were unaware of the other systemic consequences (diabetes mellitus, cardiovascular diseases, and cancers) (Table 1).

### 3.2. Sources of Vitamin D

Approximately 48% of participants incorrectly identified that sunlight that passed through glass is a source of vitamin D. Milk/dairy products (22.6%) and eggs (8.33%) were recognised as vitamin D rich foods by few students. Although many were able to identify at least one correct source (30.9%), 17% of respondents also believed that green leafy vegetables are rich in vitamin D. Adequate sunlight exposure (10 am to 2 pm with arms and leg exposed), an important source of vitamin D, was recognised by 42.4% of participants. As for adequate duration of sun exposure required for vitamin D synthesis in Indian population, only one-third (32%) of respondents were aware of it.

### 3.3. RDA and Vitamin D Supplements

About a quarter of our study population (24.6%) was aware of the recommended daily allowance (RDA) of vitamin D (600 IU) for adult men and women. Among the undergraduate students, majority (77%) had taken vitamin D supplements at some point of their life, though only few of them (24.2%) had been diagnosed with vitamin D deficiency (estimation of serum 25-hydroxyvitamin D levels) before administration.

### 3.4. Treatment of Vitamin D Deficiency

About half of the participants (52%) correctly identified calcitriol as the active form of vitamin D for biochemical functioning in the body. Cholecalciferol was thought to be the active vitamin D by 39.2% participants. More than 80% of the students were unaware of the serum levels of vitamin D indicating insufficiency/deficiency that mandates treatment. With regard to the recommended forms of vitamin D for treatment of vitamin D deficiency, a similar number of participants identified cholecalciferol (40%) and calcitriol (41.6%). Only a quarter (27.3%) of students selected correct dosage regimen (50000 IU once a week for 6–8 weeks). Many students incorrectly associated cholecalciferol (41.6%) with hypercalcemia. Also, 34.9% of the participants were aware that calcium supplementation is important in treatment of vitamin D deficiency in cases of low dietary intakes of calcium.

## 4. Discussion

The present study demonstrates gaps in basic knowledge about vitamin D, its benefits, and management of vitamin D deficiency among medical undergraduate students. Majority of the students believed that vitamin D deficiency is prevalent only in high-risk groups confined to urban areas. They were unaware that vitamin D deficiency has attained epidemic proportions across the world irrespective of age groups, populations, and geographical regions [2].

With regard to the importance of vitamin D, it is a key factor for maintenance of calcium and phosphate metabolism and bone homeostasis. In line with previous studies, majority of students (94%) correctly reported that vitamin D deficiency could lead to bone and skeletal disorders [13]. However, other population groups like office workers in Australia and women in China have been reported to be unaware of this fact, which could be attributed to their nonmedical background [17, 18]. Recent observational studies have found higher prevalence of cardiovascular diseases, type 2 diabetes mellitus, autoimmune disorders, cancer, mental disorders, and infectious diseases in vitamin D deficient states [1, 19]. However, our participants were not cognizant of these systemic associations of long-term vitamin D deficient state as reported previously in literature [20–22].

Apart from adequate sun exposure, fortified milk and other food products (salmon, cod liver oil, sundried mushrooms, and egg yolk) are rich in vitamin D. We observed that students need to have knowledge about natural, dietary, and fortified sources and that these dietary sources alone are insufficient in treatment of deficient states. In our population, as in previous studies [13, 20–23], knowledge regarding vitamin D dietary sources was insufficient. Vitamin D deficiency may be overlooked in Asian countries including India, perhaps on the assumption that vitamin D deficiency is unlikely to occur in regions that receive ample sunshine throughout the year [24–26]. Sun exposure is one of the best natural sources for prevention and treatment of vitamin D deficiency. Various sociocultural and dietary factors, limited outdoor activity due to urbanization, air pollutants, and negative attitudes towards sunlight with extensive use of sunscreens for “fairer” skin all attribute to high prevalence of vitamin D deficiency in our country [19, 20, 25–27]. In addition, it is known that sunlight that passes through glass is not suitable to build vitamin D levels [24]. Unfortunately, most medical students were unaware of this important fact. In addition, adequate sun exposure varies according to time of the day and latitude and duration of exposure in different parts of the world. In New Delhi, India, where the study was conducted, it is recommended that exposure of arms and legs to sunlight between 10 am to 2 pm for 10 min to half an hour twice a week is sufficient to achieve adequate vitamin D production in Indian skin type [24]. In line with previous studies (Vu et al.), more than 50% of our students believed that substantially greater duration of exposure (>1 to 4 hours) irrespective of the time of the day is required for vitamin D synthesis. Thus, there is a need to sensitize students to the fact that sunlight is one of the most important sources of vitamin D and stimulates the production of vitamin D3 in the skin, depending on hour of the day, duration of exposure, age, skin pigmentation, clothing style, and sunscreen use [24–26, 28, 29].

With regard to the amount of dietary intake of vitamin D, only one-third of respondents correctly identified the RDA of vitamin D. Institute of Medicine (IOM), USA, recommends RDA of 600 IU daily for adult male and females to optimise bone health. However, in absence of adequate vitamin D intake, insufficiency or deficiency may result. US Endocrine Society Classification guidelines suggest that vitamin D (25-hydroxycholecalciferol) serum levels in an adult indicate insufficiency at 20–29 ng/mL and deficiency at <20 ng/mL (Table 2). Apart from RDA for normal healthy adult, students should be made aware of how it varies according to age, sex, special states (elderly and pregnancy), and other disease conditions (renal and liver diseases). Serum vitamin D levels that indicate sufficiency and insufficiency also need to be emphasized [30, 31].

Treatment in the right form of vitamin D supplement, adequate dose, and duration is imperative to prevent long-term health consequences of vitamin D deficiency or toxicity [30–33]. It is recommended that vitamin D (ergocalciferol or cholecalciferol) should be administered in a dose of 50,000 IU per week for 8 weeks in addition to bimonthly supplements to maintain adequate vitamin D levels [34]. It is important to understand that any dosing schedule (preferably dosing on a daily basis) should allow a stable circulating concentration of vitamin D to ensure an optimal autocrine environment for vitamin D metabolism in nonbony tissues [35]. Calcitriol (1,25-dihydroxycholecalciferol) and alfacalcidol (1 alpha hydroxycholecalciferol) are not recommended as they may be associated with hypervitaminosis and hypercalcemia; requiring monitoring of serum calcium when initiated on these supplements [30, 32, 33]. Thus, these are not recommended for treatment of nutritional deficiency [33]. However, we observed a lack of awareness among students regarding the various biochemical forms, dose, and duration of vitamin D supplementation for treatment of nutritional deficiency. It is known that prolonged self-medication with high doses of vitamin D supplements, without biochemical confirmation of deficient serum levels (estimation of serum 25-hydroxyvitamin D), could pose a serious health hazard [36]. Though vitamin D toxicity is one of the rarest medical conditions, students need to be aware that adverse effects of treatment can be considerable, as nearly 52% of them had self-administered supplements without a confirmatory diagnosis of vitamin D deficiency [36].

Results of the current study are largely consistent with those conducted in other countries when it comes to identifying a knowledge deficit [13, 17, 18, 20–23]. Despite widespread attention about vitamin D deficiency, considerable gaps in knowledge of vitamin D, its sources, required intake, factors affecting levels, and associated health benefits were evident. Though students at this stage of their training understand the physiological and biochemical roles of vitamin D, its metabolism, and occurrence of deficient states in children and adults; their knowledge gap could be attributed to lack of emphasis on knowledge empowerment (educated as a part of their curriculum training on the importance of sensible sunlight exposure and dairy and nondairy products fortification) and opportunities to transfer what they have learnt to a real life practice setting. In addition, limited health awareness campaigns at the level of physicians, high-risk, and general populations [37] mandate the need for policy and public health recommendations of the government and its agencies on easily accessible reliable information for the public about vitamin D and sun exposure [20, 38, 39]. This lack of initiative is evidenced by absence of any national health policies or programmes to improve vitamin D related health (fortification of food products with vitamin D, guidelines for evaluation, treatment, and prevention of vitamin D deficiency) in our population [40]. In addition, an expert group of the Indian Government that gathered to revise and update the human nutrient requirements and the daily recommended dietary allowance for Indians concluded that outdoor physical activity is a means of not only achieving adequate vitamin D but also controlling overweight and obesity in Indian population. They felt that the recommendations (by international agencies) for obtaining vitamin D from fortification of diet and supplementation were essential only in advanced countries where exposure to sunlight is limited. Thus, only under situations of minimal exposure to sunlight, a daily supplement of 400 IU (10 *μ*g) was recommended [26, 40].

This research evidence has reinforced the need for sensitization of medical undergraduates early in their training regarding vitamin D, its importance, and prevention and treatment of deficiency. Awareness about vitamin D at this stage of medical profession would not only benefit their own health but also instill a health-related behaviour change and increased awareness and knowledge as future medical practitioners [41]. Well-aware health professionals are more likely to be vigilant about this disorder and identify and take corrective measures at the earliest to prevent long-term health consequences in the vulnerable population. Furthermore, evidence supports the importance of physician mediated knowledge translation, as people report higher rates of intention to use vitamin D when they have been informed of the benefits via physician [42]. In addition, the findings from this assessment will provide important information to determine what next steps will be necessary to promote awareness of vitamin D among medical students. It could provide baseline data to design modules for training of medical professionals that would help them in identification, prevention, and treatment of vitamin D deficiency.

Results of this study, if extrapolated to general population, could indicate a gross lack of knowledge about this worldwide pandemic and how to prevent it at an individual level. In order to facilitate health-related behaviour change, program planners need to start with an exploration of the target population's needs, which includes understanding of their knowledge about the specific issue of interest (i.e., vitamin D) [43]. It is unlikely that people would identify with and take corrective measures to resolve an issue they are unaware of. Thus, community levels awareness is essential to stop this silent outbreak. Future health promotion programmes with effective educational campaigns are needed to increase awareness about vitamin D so that they will inform and encourage people to adopt health-related behaviours (adequate intake) to decrease rates of insufficiency, especially in at-risk groups, thereby improving overall health [15, 16, 44].

The key strengths of this study include an important research question, the outcome of which could not only affect the study population, that is, medical students but indirectly also impact the general population/patients that they would treat as future medical practitioners. The results of this study will make an important contribution by defining and providing an evidence base for the existing knowledge deficit and instituting corrective measures at a crucial step of medical training. However, results may not be generalizable to all student populations as our sample was derived from a single superspeciality tertiary health care centre in India. The cross-sectional nature of our study limited subsequent assessment and translation of the imparted knowledge into practice. In addition, the questionnaire was primary developed for this study and was not used or validated beyond the context of the present study.

## 5. Conclusion

Our study highlighted a lack of awareness about the importance of vitamin D, worldwide prevalence of vitamin D deficiency, and its management among medical students. This knowledge deficit could provide baseline data to design training modules for medical professionals that would help them in identification, prevention, and treatment of vitamin D deficiency. Increased awareness at an early stage of medical training could instill adoption of health-related behaviours at personal and professional level. Effective educational campaigns targeted to specific populations would increase awareness about adequate intake of vitamin D, thereby improving overall health.

## Figures and Tables

**Table 1:** Vitamin D: percentage of student's response awareness of vitamin D.

Question	*N* (%)
Status of vitamin D deficiency in India	
Only in high risk groups	60 (23.8)
Urban population	119 (47.2)
Epidemic proportions	45 (17.8)
Rare	21 (8.3)
High risk groups for vitamin D deficiency	
Infants, pregnant, and lactating women	192 (76.1)
Elderly	15 (5.9)
Patients with diabetes mellitus	6 (2.3)
People with naturally fair skin	23 (9.1)
None of the above	16 (6.3)
Problems associated with vitamin D deficiency	
Bone and skeletal disorders	238 (94.4)
Diabetes mellitus	2 (0.7)
Cardiovascular disease	1 (0.3)
Cancer	1 (1.1)
Autoimmune disorders	4 (1.5)
None of the above	2 (0.7)
Sources of vitamin D	
Green leafy vegetables	43 (17.0)
Sunlight that passed through glass	83 (48.0)
Milk	57 (22.6)
Egg yolk	21 (8.3)
None of the above	10 (3.9)
Adequate sun exposure to achieve sufficient vitamin D levels in New Delhi	
Sun exposure (10 am–2 pm) on exposed arms and legs	107 (42.4)
Sunlight passed through glass (10 am–2 pm) on exposed arms and legs	46 (18.2)
Sunlight exposure through glass (2–4 pm) on exposed arms and legs	9 (3.5)
Sun exposure (7 am–10 am) on exposed arms and legs	79 (31.3)
None of the above	11 (4.3)
Minimum amount of sun exposure required for synthesis of vitamin D in New Delhi	
1 hour/day	87 (34.5)
30 min/twice a week	81 (32.1)
2 hours a day	51 (20.2)
4 hour/twice a week	24 (8.7)
None of the above	11 (4.3)
RDA of vitamin D	
600 IU	62 (24.6)
800 IU	84 (33.3)
1000 IU	55 (21.8)
2000 IU	13 (5.1)
Ever taken vitamin D supplement	
No	58 (23)
Yes, with estimation of serum 25-hydroxyvitamin D levels	61 (24.2)
Yes, without estimation of serum 25-hydroxyvitamin D levels	132 (52.3)
Recommended form of vitamin D supplement for nutritional deficiency	
Alfacalcidol	19 (7.5)
Cholecalciferol	101 (40.0)
Calcitriol	105 (41.6)
Either of the above	22 (8.7)
None of the above	5 (1.9)
Active biochemical form of vitamin D	
Alfacalcidol	2 (0.7)
Calcitriol	133 (52)
Cholecalciferol	99 (39.2)
Ergocalciferol	7 (2.7)
None of the above	18 (7.1)
Vitamin D serum levels in an adult indicate	
Insufficiency at 20–29 ng/mL; deficiency ≤20 ng/mL	46 (18)
Insufficiency at 10–19 ng/mL; deficiency ≤15 ng/mL	50 (19.8)
Insufficiency at 20–29 ng/mL; deficiency at ≤30 ng/mL	56 (22.2)
Insufficiency at 20–29 ng/mL; deficiency at ≤10 ng/mL	35 (13.8)
None of the above	65 (25.7)
Dose regime of vitamin D3 recommended for treatment of vitamin D deficiency	
50,000 IU of vitamin D3 once a week for 6–8 weeks	69 (27.3)
60,000 IU once a week for 8 weeks	29 (11.5)
5000 IU once a week for 10 weeks	86 (34.1)
8000 IU/day for 6 months	22 (8.7)
None of the above	46 (18.2)
Biochemical form of vitamin D most commonly associated with hypercalcemia and hypervitaminosis	
Calcitriol	84 (33.3)
Alfacalcidol	37 (14.6)
Cholecalciferol	105 (41.6)
None of the above	37 (14.6)
Are calcium supplements required for all in treatment of vitamin D deficiency	
Yes	164 (65)
No	88 (34.9)

**Table 2:** Vitamin D status in relation to 25 (OH) D^#^ levels [30, 31].

IOM (Institute of Medicine)	
Severe deficiency	<5 ng/mL
Deficiency	<15 ng/mL
Sufficiency	>20 ng/mL
Risk of toxicity	>50 ng/mL

US Endocrine Society Classification	

Deficiency	<20 ng/mL
Insufficiency	21–29 ng/mL
Sufficiency	>30 ng/mL
Toxicity	>150 ng/mL

^#^25 (OH)D = 25-hydroxycholecalciferol.
